# Biomechanical Effects of a Unilateral Approach to Minimally Invasive Lumbar Decompression

**DOI:** 10.1371/journal.pone.0092611

**Published:** 2014-03-21

**Authors:** Zachary A. Smith, Georgios A. Vastardis, Gerard Carandang, Robert M. Havey, Sean Hannon, Nader Dahdaleh, Leonard I. Voronov, Richard G. Fessler, Avinash G. Patwardhan

**Affiliations:** 1 Northwestern Feinberg School of Medicine, Department of Neurological Surgery, Northwestern University, Chicago, Illinois, United States of America; 2 Loyola University Stritch School of Medicine, Department of Orthopaedic Surgery, Maywood, Illinois, United States of America; 3 Edward Hines Jr. VA Hospital, Hines, Illinois, United States of America; Georgia Regents University, United States of America

## Abstract

Minimally invasive (MI) lumbar decompression became a common approach to treat lumbar stenosis. This approach may potentially mitigate postoperative increases in segmental motion. The goal of this study was to evaluate modifications to segmental motion in the lumbar spine following a MI unilateral approach as compared to traditional facet-sparing and non-facet sparing decompressions. Six human lumbar cadaveric specimens were used. Each specimen was tested in flexion-extension 0 N and 400 N of follower preload), axial rotation, and lateral bending. Each testing condition was evaluated following three separate interventions at L4–L5: 1) Minimally invasive decompression, 2) Facet-sparing, bilateral decompression, and 3) Bilateral decompression with a wide facetectomy. Range of motion following each testing condition was compared to intact specimens. Both MI and traditional decompression procedures create significant increases in ROM in all modes of loading. However, when compared to the MI approach, traditional decompression produces significantly larger increase in ROM in flexion-extension (p<0.005) and axial rotation (p<0.05). It additionally creates increased ROM with lateral bending on the approach side (p<0.05). Lateral bending on the non-approach side is not significantly changed. Lastly, wide medial facet removal (40% to 50%) causes significant hypermobility, especially in axial rotation. While both MI and traditional lumbar decompressions may increase post-operative ROM in all conditions, a MI approach causes significantly smaller increase in ROM. With an MI approach, increased movement with lateral bending is only toward the approach side. Further, non-facet sparing decompression is further destabilizing in all loading modes.

## Introduction

Minimally invasive spine surgery (MISS) techniques are being increasingly utilized in practice.[1], [2], [3], [4], [5], [6], [7], [8], [9] Of these MISS techniques, the minimally invasive decompression of lumbar stenosis is both one of the most frequently utilized and “mature” techniques.[3], [7] Favorable clinical outcomes have been reported with this decompression technique, even in more challenging patient populations [10], [11]. The most prominent of its advantages is a decrease in iatrogenic soft-tissue injury, a difference that is often reflected in peri-operative outcome measures [10], [12], [13].

The potential biomechanical implications of this surgical approach are less understood. While there are several described variants, the focus of this report is that of a unilateral approach to single-level bilateral lumbar decompression. This technique utilizes sequential tubular muscle dilation and either a microscope or endoscope. There are two anatomic features of this technique that may help to maintain native biomechanics [14]. Firstly, the trajectory of decompression allows for an internal decompression. This requires only a partial *ipsilateral* facetectomy and preserves the contralateral facet. As the facet joints are critical to lumbar stability, especially in axial rotation, this may minimize post-operative instability. Secondly, the spinous processes and the midline ligaments are well preserved. When these midline structures are removed, this may negatively impact native spinal biomechanics.

The goal of this study is to evaluate the biomechanical effects of a modern technique for minimally invasive lumbar decompression. Previous studies have evaluated the role of graded facet removal [15], [16]; however, to our knowledge, a minimally invasive model using complete lumbar spines has not been investigated. In this biomechanical cadaver study, we evaluated the effects of minimally invasive decompression (MI-D) on native lumbar spine mechanics.

## Materials and Methods

### Specimens and Experimental Setup

Six fresh-frozen human cadaveric lumbar spine specimens (L1-sacrum, age 56.5±15.7, 4 males, 2 females) were used for this study (Table 1). Specimens were procured from the GenLife Institute tissue bank (Phoenix, AZ).

**Table 1:** Specimen demographics for six lumbar spine segments.

Specimen	Age (years)	Sex	Cause of Death
1	73	M	Lung Cancer
2	79	M	Lung Cancer
3	41	M	Cardiac Arrest
4	44	M	GSW to the Head
5	52	F	Colon Cancer
6	50	F	Breast Cancer

Abbreviations:

M =  Male, F =  Female.

GSW  =  Gun shot wound.

Radiographic screening was performed to exclude specimens with fractures, metastatic disease and bridging osteophytes. The specimens were thawed and stripped of the paraspinal musculature while preserving the discs, facet joints, and osteoligamentous structures. All tests were performed at room temperature.

**Figure 1:**
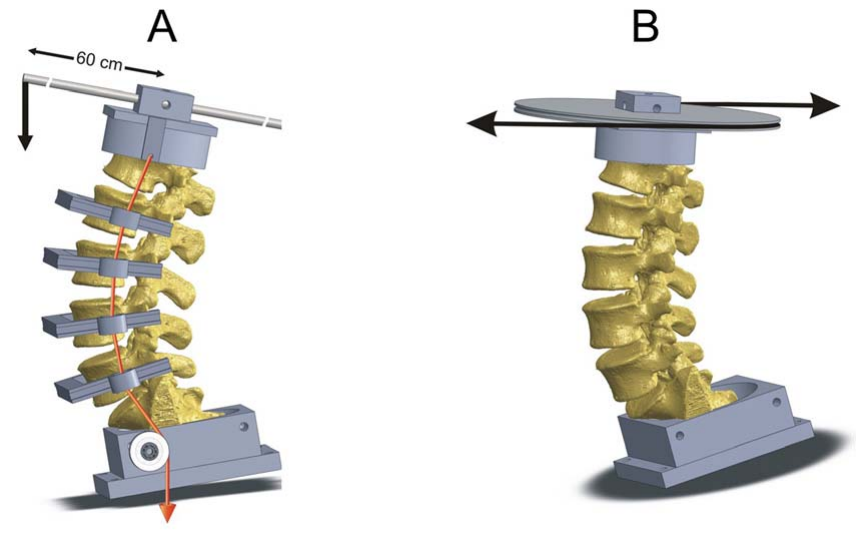
Schematic of the loading apparatus for range of motion tests in flexion-extension and axial rotation. Testing for ROM in lateral bending was performed using an offset-loading arm in the frontal plane.

Specimens were fixed to the apparatus at the caudal end and free to move in any plane at the proximal end. A moment was applied by applying an offset vertical load for flexion, extension, and lateral bending. Axial rotation was produced by applying two equal and opposite forces (**Fig. 1**). The specimen was cycled between specified maximum moment endpoints in flexion, extension, lateral bending, and axial rotation.

The angular motion of the L1 to L5 vertebrae relative to Sacrum was measured using an optoelectronic motion measurement system (Model Certus, Optotrak, Northern Digital, Waterloo, Ontario). In addition, bi-axial angle sensors (Model 902-45, Applied Geomechanics, Santa Cruz, CA) were mounted on each vertebra to allow real-time feedback for the optimization of the preload path. A six-component load cell (Model MC3A-6-1000, AMTI Inc., Newton, MA) was placed under the specimen to measure the applied compressive preload and moments. Fluoroscopic imaging (GE OEC 9800 Plus digital fluoroscopy machine) was used to document implant position.

The follower load technique was used to apply compressive preloads to the lumbar spine during the range of motion experiments in flexion and extension [17]. The compressive preload was applied along a path that follows the lordotic curve of the lumbar spine. By applying a compressive load along the follower load path the segmental bending moments and shear forces due to the preload application were minimized. This allowed the lumbar spine to support physiologic compressive preloads without damage or instability.

The follower load cable guides were attached to the vertebral bodies of L2–L5. The preload was applied using bilateral loading cables that were attached to the cup holding L1 vertebra. The cables passed freely through guides anchored to each vertebra and were connected to a loading hanger under the specimen. The cable guide mounts allowed anterior-posterior adjustments of the follower load path within a range of about ten millimeters. The preload path was optimized by adjusting the cable guides to minimize changes in lumbar lordosis when a compressive load of up to 400 N was applied to the specimen beginning in its neutral posture. Our previous experience demonstrated that the optimization of the follower preload path minimizes the effects of artifact moment and shear force on the range of motion of the spine in flexion-extension.

### Experimental Protocol

All specimens were tested in each of the three conditions: intact, following minimally invasive surgery, and after open laminectomy. Each condition was tested under moments of +8 Nm in flexion and −6 Nm in extension with compressive follower preloads of 0 N and 400 N. Previous biomechanical studies on human lumbar spine specimens have used moments ranging from 5 Nm to 10 Nm, depending on the specific goals of their experimental studies.^18^ In our experience, maximum moments of 8 Nm in flexion and 6 Nm in extension are generally adequate to capture the vast majority of the available ROM of the cadaveric lumbar spine specimen without the risk of causing tissue damage. This is particularly important if multiple test runs on the same specimen are planned, as in the case of the present study. The load-displacement data were first collected on the intact specimen until two reproducible loops were obtained for each loading case. This was then repeated after each surgical technique. This protocol was also performed for lateral-bending (±6 Nm) and axial rotation (±5 Nm).

### Surgical Techniques

Three testing conditions were compared to the intact condition. A minimally invasive L4/L5 decompression was patterned after a minimally invasive unilateral approach for bilateral internal decompression, which can be aided by either microscope or endoscope. A 18 mm×6 cm METRx portal was docked (Figure 1) from a left-approach at L4/L5. A unilateral L4 hemilaminotomy with a partial superior L5 hemilaminotomy was completed through the METRx tube, using standard operative tools, including a hand-held drill. The ligamentum flavum was completely removed and bilateral L4/L5 foramina were opened. Confirmation of a “pedicle-to-pedicle” decompression, including an evaluation of the contralateral foramina, was undertaken with a lateral fluoroscopy (Figure 1).

**Figure 2:**
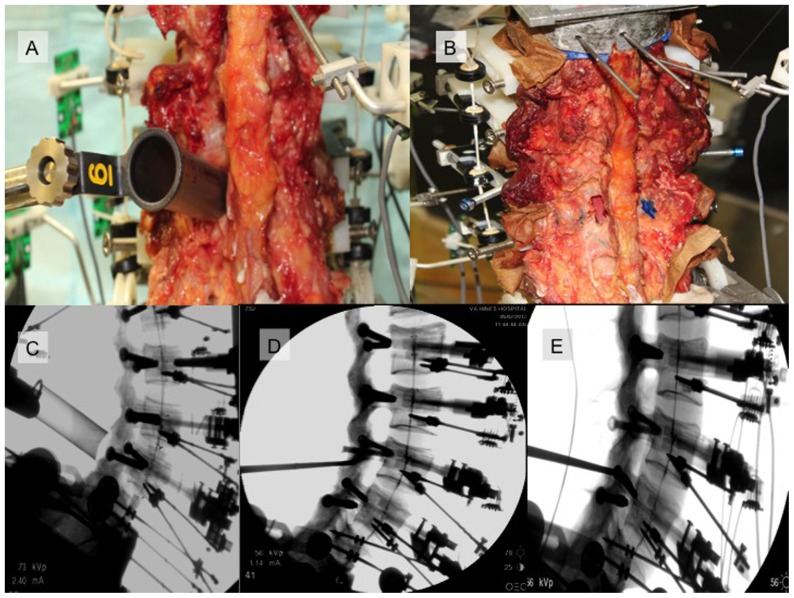
Images demonstrating testing of the minimally invasive surgical procedure. Unilateral docking of the METRx tube at L4/L5 is shown (A) as well as post-procedural anatomic changes following decompression (B). A radiograph demonstrating tubular docking is shown in C. Following complete removal of the ligamentum flavum and internal bony decompression, a curette was placed at the superior and inferior extent of decompression to confirm a “pedicle-to-pedicle” dural decompression.

The second surgical condition patterned a standard midline L4/L5 decompression with an 80% removal of the L4 lamina and 20% removal of the superior portion of L5. The supraspinous and intraspinous ligaments were cut and the L4 spinous process removed. For both minimally invasive and traditional decompressions, facet removal was carefully checked. Two independent observers marked and confirmed the medial (most medial facet fiber attachments) and lateral extent of the facets. For the first two testing conditions, 15–20% of medial facet was removed. Markings of these facet lines are shown in Figure 2.

**Figure 3:**
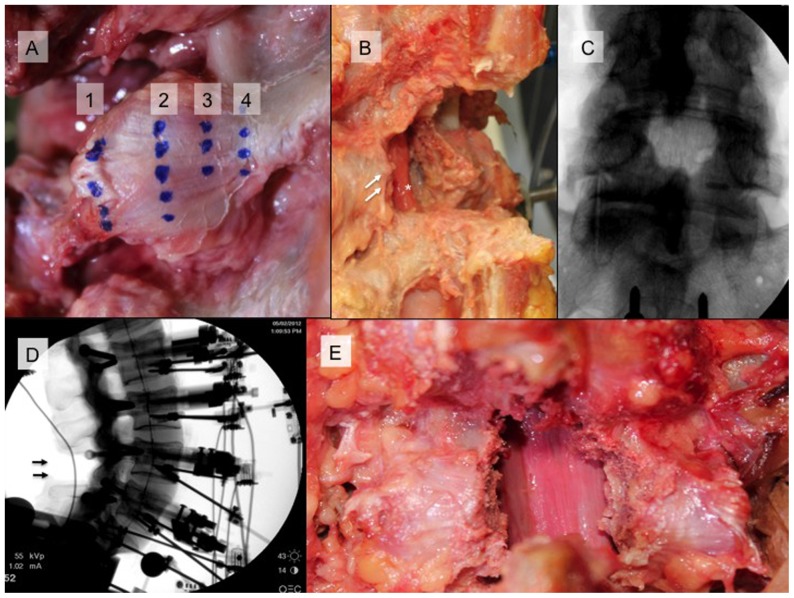
Capsular facet fibers were marked in each specimen by two observers. As shown in A, Line 1 and 4 correspond to the lateral and medial facet extent with line 2 the midpoint and 3 at 25% distance from midline. An oblique view in B, shows “traditional open L4/L5 decompression with extent of facet removal (double arrows) and dural decompression (*) shown). AP and lateral radiographs of this decompression are in C and D. Image E represents “wide factectomy” of 40% bilateral medial facet removal.

The final testing condition was a “wide” decompression (Figure 2 and 3). The second operative condition was extended to involve a 20% greater removal of the medial facets (40% resection). The capsule of the facet was only disrupted to this line and the midline capsule was undisturbed.

### Experimental Protocol

Analysis of variance (ANOVA) was used to assess the effects of various surgical steps on the range of motion in flexion-extension, left and right lateral bending, and left and right axial rotation. Where indicated by the results of the ANOVA, statistical analyses with one-tailed Bonferroni-adjusted p values were used to evaluate significant differences in ROM between each testing condition.

## Results

Flexion and extension ROM values for each surgical condition are reported for testing with and without a 400 N follower preload (Table 2). In general, the effect of the follower preload was to attenuate post-operative hypermobility. Given the stabilizing forces of the muscles *in vivo*, this would be expected.

**Table 2:** L4–L5 range of motion (in degrees), corresponding to a moment of 8 Nm in flexion and 6 Nm in extension, ±6 Nm in lateral bending, and ±5 Nm in axial rotation.

Testing Condition				
	Intact	MI-D	TD	WTD
Total FE (no follower preload)	9.4 (1.3)	10.2* (1.4)	11.2*† (1.2)	11.2*† (1.2)
Total FE with 400 N follower preload	9.2 (1.7)	9.6* (1.9)	10.7*† (1.9)	12.3*† (1.9)
Flexion (400 N follower preload)	6.1 (1.8)	6.3 (1.7)	6.7* (1.6)	8.1*†(0.8)
Extension (400 N follower preload)	3.0 (0.8)	3.3 (0.8)	4.0*† (1.0)	4.3* (1.4)
Total Axial Rotation	3.7 (1.3)	4.0* (1.4)	4.5*† (1.5)	6.3*† (2.5)
Left Axial Rotation	1.7 (0.6)	1.9* (0.8)	2.3*† (0.9)	3.3* (1.4)
Right Axial Rotation	2.0 (0.7)	2.1 (0.7)	2.2* (0.8)	3.0† (1.1)
Total Lateral Bending	8.0 (2.8)	8.4* (2.8)	8.6* (2.7)	10.4*† (1.9)
Left Lateral Bending	4.0 (1.3)	4.3* (1.4)	4.4† (1.2)	5.4*† (1.2)
Right Lateral Bending	3.9 (1.6)	4.0 (1.5)	4.2* (1.6)	5.0 (0.7)

Abbreviations:

FE  =  Flexion-Extention.

MI –D =  Minimally invasive decompression.

TD  =  Traditional decompression.

WTD  =  Wide traditional decompression.

(SD)  =  Standard deviation.

(*denotes statistically significant difference from the intact condition;

† denotes statistically significant difference from the MI-D condition).

The values in parentheses denote one standard deviation of the mean.

A traditional decompression produced significant differences in range of motion in flexion-extension (p<0.01), lateral bending (p<0.05) and axial rotation (p<0.01) (Table 2). It should be noted that a MI-decompression also produced significant ROM changes in each of these same conditions (p<0.05); however, the traditional decompression creates significantly more mobility as compared to MI-D at L4/L5 in flexion-extension (p<0.01) and axial rotation (p<0.05) (Table 2).

When the effect of MI-D was evaluated separately for left and right lateral bending, the ROM increase, as compared to the intact condition, was isolated to bending toward the side of the approach (p<0.05). Lateral bending to the opposite direction was not significant (p>0.05).

As mentioned in the Surgical Techniques section, less than 25% of the medial facet was removed in the initial two testing conditions. However, we found that a more extensive resection of the facet (40% resection) resulted in additional ROM increases that were significant (Table 2). This effect was most evident in axial rotation.

## Discussion

While the technique of minimally invasive lumbar decompression has been well described [12], the effect of this approach on spinal biomechanics remains poorly understood. It is quite difficult to evaluate how this surgical approach, as well as other techniques, impacts the spine *in vivo*. As such, the testing of a cadaveric model that represents the anatomic changes enacted by this surgical approach may help us better understand how this surgery changes native spinal biomechanics. In this study, we have evaluated a cadaveric lumbar spine following minimally invasive decompression.

In general, the lumbar spine specimens used in this study were without evidence of significant degenerative disease and adequate bone quality. Each full lumbar spine segment was first tested intact with a follower preload, to simulate *in vivo* conditions with load-bearing musculature, and then after each surgical condition. The results of this study demonstrate that a minimally invasive approach produces a significantly smaller increase in segmental spine motion in flexion-extension and axial rotation when compared to a traditional, midline laminectomy. In right lateral bending, there was a significantly smaller increase in motion from intact after minimally invasive surgery as compared to traditional decompression. However, with lateral bending to the side of the surgical approach (in our case, from the left), both approaches were equally destabilizing.

From our results, it also seems that even partial facet removal can have implications upon post-operative spinal mobility. With the MI approach, even removal of less than 15–25% of the facet can instigate mild hypermobility with lateral bending to the approach side. However, lateral bending to the contralateral side is not affected. This implies that the MI approach may preserve the biomechanics of the opposite facet. Further, our results further validate previous reports demonstrating that graded removal of bilateral facets will result in predictable post-operative hypermobility, especially with axial rotation. Abumi, et al. [15] demonstrated significant instability created by unilateral complete facet removal. Our model differs, as we tested a full lumbar segment with a follower preload; however, our results show similar effects from aggressive facet removal.

In the clinical setting, increased segmental mobility may influence clinical outcomes. While decompressive laminectomy is generally a highly successful operation, clinical outcomes commonly deteriorate with longer follow-ups [18], [19], [20]. As reported in by Iguchi et al. [21], sustained long-term outcomes following non-instrumented decompression may be limited by post-operative spinal instability. This finding was especially prominent in patients with evidence of pre-operative instability. Further, the incidence of lumbar spondylolisthesis following laminectomy, even with facet preserving techniques, has been reported from 8% to 31% [22], [23]. These clinical findings parallel reported data from *in vitro* models. Bisschop et al. recently demonstrated that following single-level laminectomy, there was a significant decrease in both segmental shear stiffness as well as shear-yield force [24], [25].

Clinically significant spinal stenosis is more common to older patients. Thus, pre-operative risk factors, such as medical co-morbidities, increased body habitus, and underlying degenerative spinal changes (including segmental instability) are more common in surgical candidates for lumbar decompression. Uniquely, these patients may be the ones who benefit most from minimally invasive techniques. Rosen et al. found excellent clinical outcomes, with few complications, in a study of 57 patients over 75 years of age, treated with MI lumbar decompression [11]. This same finding has been shown in obese patients with both instrumented [26] and non-instrumented techniques.^28^ Many reports have questioned the role of non-instrumented decompression in patients with pre-operative spondylolithesis. However, in a recent report on minimally invasive lumbar decompression, only a single case (1.8%) in 54 treated patients required subsequent spinal fusion. This was in spite of 27 patients (50%) having a grade I (or greater) spondylolisthesis at baseline [10].

There are multiple modern variants of MI lumbar decompression. Minimally invasive techniques have been described using a microscope [27], [28] or an endoscope [12], [13]. While we evaluated a unilateral approach to bilateral decompression in this study, surgical approaches utilizing bilateral hemilaminotomies have also been described. Fundamental to each of these approaches is preservation of the midline bones and ligaments and judicious medial facetectomy. We feel that both approaches allow for an adequate decompression of the dura. However, a potential limitation of our work is that we have not studied the bilateral approach in our biomechanical model.

A notable limitation in our study is that we used only one minimally invasive technique for decompression. For instance, Lee et. al studied a bilateral laminotomy technique that preserves the midline osteo-ligamentous complex [29]. In their cadaveric simulation model, they a significant decrease in motion for this simulated surgery when compared to a traditional laminectomy. Although there are differences between the decompression in our study and that of Lee et.al, we believe that the preservation of the midline ligamentous complex is a shared component of successful minimally invasive lumbar decompressions. As can be seen in clinical practice, technical variants can be applied equally with success and less injury to the native spinal anatomy.

The methodology of our study also presents other limitations. For instance, cadaveric studies cannot account for the effect of muscular forces on each spinal segment. However, the implementation of full lumbar segments using the follower preload technique, which serves to simulate these forces, may minimize this limitation [17]. In addition, our study primarily used younger specimens. Although this is ideal for studying native spinal biomechanics, this may not model the patients who are commonly candidates for surgery.

## Conclusions

1. A minimally invasive, unilateral approach to treat lumbar stenosis produces significantly less biomechanical instability than a traditional midline laminectomy, in all modes of testing.
2. MI decompression does increase ROM in most testing conditions.
3. Lateral bending and axial rotation are only affected on the side of the approach with MI decompression.
4. Wide bilateral facetectomy, even when comprising <50% of the facet, is progressively destabilizing, especially in axial rotation.
